# A novel variant in *GAS2* is associated with autosomal dominant nonsyndromic hearing impairment in a Chinese family

**DOI:** 10.1186/s40246-024-00628-2

**Published:** 2024-07-02

**Authors:** Luping Zhang, Danya Zheng, Lian Xu, Han Wang, Shuqiang Zhang, Jianhua Shi, Nana Jin

**Affiliations:** Institute for Translational Neuroscience, Department of Otolaryngology-Head and Neck Surgery, The Second Affiliated Hospital of Nantong University, Affiliated Hospital of Nantong University, Nantong University, Nantong, Jiangsu 226001 China

**Keywords:** *GAS2*, Autosomal dominant nonsyndromic hearing loss, Protein degradation, Microtubule, Apoptosis

## Abstract

Knockout of *GAS2* (growth arrest-specific protein 2), causes disorganization and destabilization of microtubule bundles in supporting cells of the cochlear duct, leading to hearing loss in vivo. However, the molecular mechanism through which *GAS2* variant results in hearing loss remains unknown. By Whole-exome sequencing, we identified a novel heterozygous splicing variant in *GAS2* (c.616–2 A > G) as the only candidate mutation segregating with late-onset and progressive nonsyndromic hearing loss (NSHL) in a large dominant family. This splicing mutation causes an intron retention and produces a C-terminal truncated protein (named GAS2mu). Mechanistically, the degradation of GAS2mu via the ubiquitin-proteasome pathway is enhanced, and cells expressing GAS2mu exhibit disorganized microtubule bundles. Additionally, GAS2mu further promotes apoptosis by increasing the Bcl-xS/Bcl-xL ratio instead of through the p53-dependent pathway as wild-type GAS2 does, indicating that GAS2mu acts as a toxic molecule to exacerbate apoptosis. Our findings demonstrate that this novel variant of *GAS2* promotes its own protein degradation, microtubule disorganization and cellular apoptosis, leading to hearing loss in carriers. This study expands the spectrum of *GAS2* variants and elucidates the underlying pathogenic mechanisms, providing a foundation for future investigations of new therapeutic strategies to prevent *GAS2*-associated progressive hearing loss.

**Supplementary Information:**

The online version contains supplementary material available at 10.1186/s40246-024-00628-2.

## Background

Hereditary hearing loss (HL), the most common congenital sensory defect, is caused by genetic and/or non-genetic factors [8, 11]. Based on the clinical manifestations, hereditary hearing loss can be classified as syndromic (< 30% of cases) or nonsyndromic (> 70% of cases). The presence of only one dominant allele of the disease gene on the autosomal chromosome can cause autosomal dominant nonsyndromic hearing loss (ADNSHL), which accounts for approximately 20% of cases of nonsyndromic hearing loss [12]. ADNSHL typically manifests with late onset and tends to be less severe than other types of hearing loss [12]. Approximately 63 causative genes for ADNSHL have been recorded in the Hereditary Hearing Loss Homepage database (http://hereditaryhearingloss.org). Among these, mutations in *MYO6*, *TECTA*, *POU4F3*, and *KCNQ4* are associated with the most prevalent forms of ADNSHL. Mutations in deafness genes frequently lead to dysfunction of hair cells and synapses, cochlear supporting cells, and/or cells in the stria vascularis and lateral wall [25]. Identifying crucial pathogenic variants associated with hearing loss in affected families, such as the well-known deafness genes *MYO6* and *KCNQ4*, and delineating the underlying mechanisms are fundamental steps in the development of gene therapy, genetic etiology and genetic counselling [7, 16, 20, 21, 25].

Growth arrest-specific protein 2 (GAS2), encoded by *GAS2*, is a cytoskeletal regulatory protein [26]. It consists of a calponin homology (CH) domain at the N-terminus and a growth arrest-specific 2-related (GAR) domain at the C-terminus, which mediate the binding of GAS2 to actin filaments and microtubules, respectively. GAS2 is expressed mainly in tissues of the liver, pancreas and thymus [26]. GAS2 is implicated in the regulation of the cell cycle and apoptosis and plays an important role in various cancers. It induces cell cycle arrest by suppressing the G1-to-S transition [29]. GAS2 serves as a proapoptotic factor that increases susceptibility to p53-dependent apoptosis under stress conditions in various cell lines [1, 2, 4]. Furthermore, GAS2 is tightly associated with increased apoptosis in vivo [10, 22]. Recently, it was reported that GAS2 is expressed in cochlear supporting cells, Pillar cells and Deiters’ cells and maintains the stiffness properties of the cells for the propagation and amplification of traveling waves through the cochlear partition in response to sound [5]. The function of outer hair cells and their vibratory responses to sound were found to be impaired in GAS2-null mice. The homozygous c.723 + 1G > A variant in *GAS2* was shown to cosegregate with autosomal recessive NSHL in one family of Somalian descent [5]. However, the effect of this novel *GAS2* variant on GAS2 expression and function is still unknown.

In the present study, we reported a novel heterozygous *GAS2* variant in a Han Chinese family that segregated with ADNSHL and performed a functional exploration of this variant. We found that the truncated GAS2 mutant (GAS2mu) protein resulted in a decrease in its own protein stability, cytoskeletal abnormalities and cellular apoptosis. Our study not only expands the spectrum of *GAS2* variants but also clarifies the underlying pathogenic mechanisms of these variants. The findings provide a foundation for future investigations into new therapeutic strategies aimed at preventing progressive hearing loss associated with *GAS2*.

## Materials and methods

### Patient clinical and audiometric data

Written informed consent was provided by all participating individuals. This study was approved by the informed consent of the subjects and the Ethics Committee of the Affiliated Hospital of Nantong University (2022-L111). All subjects underwent a comprehensive auditory evaluation, including pure tone audiometry (PTA), otoscopic examination and temporal bone high-resolution CT scanning. A family history was obtained and a general physical examination was performed to exclude individuals with possible syndromic hearing loss.

### Whole-exome sequencing (WES) and verification of the pathogenic variants

**Fig. 1:**
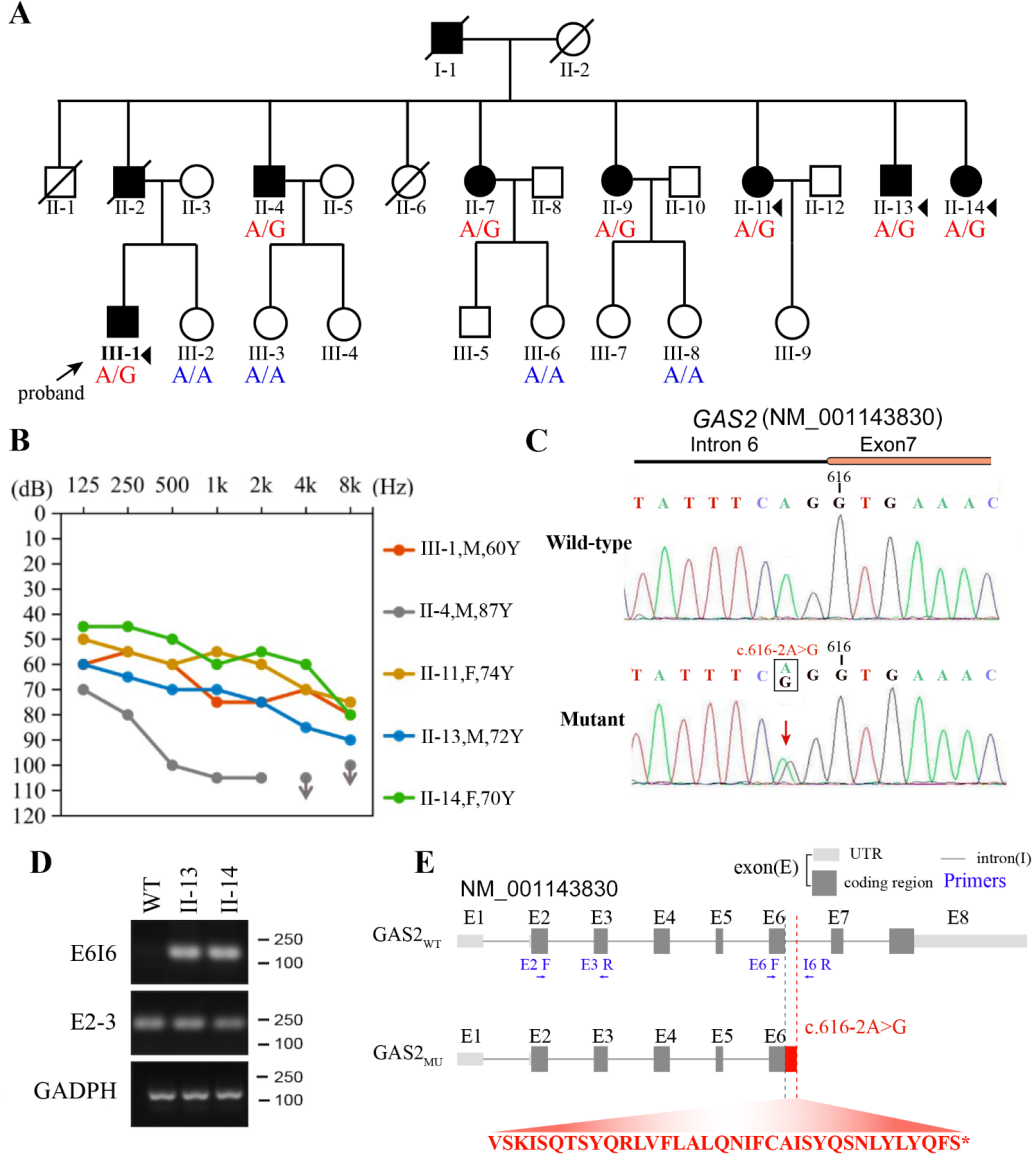
Pedigree, genotype and sequence analysis of Family NT33. (**A**)Pedigree of Family NT33. The individuals selected for WES are indicated with black triangles. The arrow indicates the proband. (**B**) Representative audiograms of members with hearing loss of Family NT33. (**C**) Sanger sequencing electropherograms of the wild-type and mutant sequences. (**D**) Agarose gel electrophoresis of cDNA amplified by PCR from RNA extracted from blood samples of family members. (**E**) Schematic representation of the GAS2 transcript and the effect of the variant on intron 6 splicing. Retention of intron 6 resulting from the GAS2 c.616–2 A > G substitution results in a stop codon (red asterisk) downstream of GAS2 exon 6

Genomic DNA was isolated from peripheral blood samples using a Blood DNA Kit (Tiangen Biotech, China). To identify the potential pathogenic variants from the sequencing data, we performed stepwise genetic analysis as previously described [24, 28]. Exome sequencing was conducted on the proband (III-1) and three other patients (II-11, II-13 and II-14) in this family (marked with triangles in Fig. 1A). Comprehensive sequencing data were analyzed based on the modes of autosomal dominant (AD) inheritance. Single-nucleotide variants and indels were filtered against the reference population databases, including the 1000 Genomes Project, Exome Aggregation Consortium (ExAC) database and the Genome Aggregation (gnomAD) Database, with a maximal allele frequency of 0.1% in AD. Bioinformatics prediction tools, including SIFT, Polyphen2, REVEL and MutationTaster, were employed. To further validate the putative pathogenic variants, we performed Sanger sequencing for the other affected family members (II-4, II-7 and II-9) and for unaffected family members (III-2, III-3, III-6, and III-8) (Fig. 1A). The primers of *GAS2* used for Sanger sequencing were: forward: 5’-TGCAGTCATTTGCCTTCAGA-3’ and reverse: 5’-CACCATATGGAAGTTCCTGCT-3’. The potential effect of this mutation on splicing was predicted using SpliceSiteFinder, MaxEntScan, NNSPLICE and GeneSplicer in Alamut Visual (version 2.13, Interactive Biosoftware, Rouen, France). The splice site scores (over 5%) was regarded as significant.

### Plasmids and antibodies

The DNA sequences encoding wild-type GAS2 (GAS2wt) and the truncated GAS2mu (containing exons 1–6 (205 a.a.) and intron 6 (36 a.a.)) were cloned and inserted into pCI/neo with an N-terminal HA tag by GENEWIZ (China). Monoclonal and polyclonal anti-HA antibodies were purchased from Sigma (St. Louis, MO, USA). Polyclonal anti-α-tubulin and monoclonal anti-β-actin antibodies were purchased from Proteintech (Wuhan, China). A polyclonal anti-Bcl-xS antibody was purchased from Thermo Fisher Scientific (Rockford, IL, USA). Polyclonal anti-P53, anti-cleaved caspase 3 (c-caspase 3) and anti-GAPDH antibodies were purchased from Cell Signaling Technology (Danvers, MA, USA). A monoclonal anti-Bcl-xL antibody was purchased from Santa Cruz Biotechnology (Dallas, Texas, USA). Horseradish peroxidase (HRP)-conjugated anti-mouse and anti-rabbit IgG were obtained from Jackson ImmunoResearch Laboratories (West Grove, PA). An ECL SuperSignal™ West Pico PLUS Kit was obtained from Thermo Fisher Scientific (Rockford, IL, USA).

### Cell culture and transfection

HEK-293T and HeLa cells were cultured in Dulbecco’s modified Eagle’s medium (DMEM) (Invitrogen, Carlsbad, CA, USA) supplemented with 10% fetal bovine serum (FBS) at 37 °C in 5% CO_2_. All transfections were performed in triplicate with Lipofectamine 3000 (Invitrogen, Carlsbad, CA, USA) and X-tremeGENE HP DNA Transfection Reagent (Roche, St. Louis, MO, USA) according to the manufacturers’ instructions. For serum starvation, the complete culture medium was replaced with FBS-free medium for 24 h after transfection. For the protein degradation inhibition experiment, 20 µM MG132 (a ubiquitin‒proteasome inhibitor; Sigma‒Aldrich) or 10 mM 3-methyladenine (3-MA; an autophagy‒lysosome inhibitor; Sigma‒Aldrich) was added to the culture medium for 12 h before harvesting the cells.

### Western blot analysis

HEK-293T cells transfected with the pCI/HA-GAS2wt or the pCI/HA-GAS2mu overexpression vector were harvested. The protein concentration was measured with a Modified Lowry Protein Assay Kit (Thermo Fisher Scientific). Equal amounts of protein from each sample were loaded onto sodium dodecyl sulfate (SDS)–polyacrylamide gel electrophoresis (PAGE) gels for separation, and the separated proteins were electroblotted onto a polyvinylidene fluoride (PVDF) membrane. The membrane was blocked with 5% fat-free milk and incubated with primary antibodies, such as anti-α-tubulin (1:1000), anti-GAPDH (1:2000), anti-HA (1:1000), anti-p53 (1:2000), anti-c-caspase3 (1:2000) and anti-β-actin (1:2000), in 5% fat-free milk with 0.1% NaN3 overnight at room temperature. After washing with TBST (Tris-HCl, pH 7.4; 150 mM NaCl; 0.05% Tween 20) three times, the membrane was incubated with the corresponding HRP-conjugated secondary antibody for 2 h. After three washes with TBST, signals on the membrane were visualized by enhanced chemiluminescence and quantified via densitometry using Multi Gauge V2.3 software (Fujifilm, Japan).

### Cell viability assay

A Cell Counting Kit-8 (CCK8; Dojindo, Japan) was used to examine cell viability according to the manufacturer’s instructions. In brief, HEK-293T cells were seeded at a density of 5000 cells/well in 96-well plates in triplicate and transfected with the indicated plasmids. After 48 h, 10 µL of CCK8 solution (Sigma, St. Louis, USA) was added to each well, and the plate was incubated for 1 h. The optical density (OD) was measured at 450 nm using a Synergy 4 plate reader (BioTek, Vermont, USA).

### TUNEL assay

The DeadEnd™ Fluorometric TUNEL System (Roche) was used to detect and quantify apoptosis. HEK-293T cells transfected with the indicated plasmids were incubated with TUNEL reaction mixture at 37°C for 1 h in a humidified atmosphere. After the cells were rinsed with PBS three times, nuclei were counterstained with 4’,6-diamidino-2-phenylindole (DAPI) staining solution. TUNEL-positive cells, with intense green nuclear staining, were identified as apoptotic cells. Images were acquired with a TCS-SP2 confocal microscope (Leica, Bensheim, Germany).

### Immunofluorescence staining

HeLa cells were plated on glass coverslips in 24-well plates and transfected with pCI/HA-GAS2wt or pCI/HA-GAS2mu. Two days later, the cells were washed with PBS and fixed with 4% paraformaldehyde in PBS for 30 min at room temperature. After washing with PBS, the cells were blocked with 10% goat serum in PBS for 1 h at 37 °C and incubated with a mouse anti-HA (1:2000) and/or a rabbit α-tubulin (1:2000) antibody overnight at 4 °C. After washing and incubation with secondary antibodies and Hoechst 33,342 at room temperature, the cells were washed with PBS, mounted with SlowFade® Gold Antifade Mountant (Invitrogen, Carlsbad, CA, USA), and visualized with a TCS-SP2 confocal microscope (Leica, Bensheim, Germany). The fluorescence intensities of cell nuclei and the whole cells were measured with ImageJ.

### Quantification of GAS2 intron 6 splicing by reverse transcription–PCR (RT-PCR)

Total intracellular RNA was extracted from 1 mg of fresh human blood with an E.Z.N.A. Blood RNA Kit (Omega Bio-tek, GA, USA) according to the manufacturer’s instructions. Total cellular RNA was isolated from cultured cells by using an RNeasy Mini Kit (QIAGEN, GmbH, Germany). Equal amounts of total RNA were used for first-strand cDNA synthesis with oligo(dT)15–18 by using an Omniscript Reverse Transcription Kit (QIAGEN, GmbH, Germany). PCR was performed with PrimeSTARTM HS DNA Polymerase (Takara Bio, Inc., Otsu, Shiga, Japan) and the following primers: exon 2, 3 (E2-3): 5’GGTGCCTTGCTCTGTCAACT3’ and 5’GAGGACCAAACCTTCCGATT3’; E6I6: 5’AGTACAGGAAACTTACTGGATG3’ and 5’TTAGACTGATACGAGATTGCA3’; and GAPDH: 5’GGTGGTCTCCTCTGACTTCAACA3’ and 5’GTTGCTGTAGCCAAATTCGTTGT3’. The GAS2 mRNA expression level was measured with the following thermal cycling conditions: 33 cycles at 98 ºC for 3 min, 98 °C for 10 s and 68 °C for 40 s, followed by a final extension step at 68 °C for 10 min. The PCR products were then separated on 1.5% agarose gels and quantitated using a Molecular Imager system (Bio-Rad, Hercules, CA, USA).

### Statistical analysis

GraphPad Prism 8.0 software was used for statistical analysis. The data are presented as the means ± SDs. For multiple-group comparisons, the data were compared by one-way ANOVA with the Bonferroni correction. For two-group comparisons, the data were compared by unpaired two-tailed Student’s t test.

## Results

### A novel *GAS2* variant is identified and found to cause hearing loss in a Chinese family

We identified a novel heterozygous mutation in *GAS2* segregating with nonsyndromic hearing loss as a probable cause for hearing impairment in a dominant family (designated Family NT33). This family spanned three generations, and at least nine family members were affected by adulthood-onset hearing impairment (Fig. 1A). In addition to a general physical examination, we also performed PTA on all participants. The unaffected subjects exhibited normal hearing function whereas all the affected subjects exhibited nonsyndromic, bilateral, progressive hearing loss, which was most predominant at high frequencies (Fig. 1B and Fig. S1). WES was performed using DNA isolated from blood samples of four affected individuals (II-11, II-13, II-14 and III-1), and a total of three candidate variants were identified: *GAS2* (NM_001143830; c.616–2 A > G), *ADAM11* (NM_002390; c.1451T > C), and *CSE1L* (NM_001316; c.250 A > G). These three variants were further genotyped in seven affected family members and four unaffected family members by the Sanger sequencing. Only a splicing variant (c.616–2 A > G in *GAS2*) segregating with hearing loss in the Family NT33 was identified (Fig. 1C). To further investigate the effect of this splicing variant, RT-PCR was performed on RNA isolated from blood samples of the affected family members (II-13 and II-14) and an unaffected subject (WT genotype). As expected, we observed retention of intron 6 of *GAS2* in the aberrantly spliced transcript in the affected family members (II-13 and II-14), as indicated by the DNA band generated by PCR amplification with the E6I6 (exon 6-intron 6) primer pair, in which one primer targets a location in intron 6 (Fig. 1D and E). The retention of intron 6 in the *GAS2* variant transcript resulted in the formation of a new open reading frame and an in-frame stop codon in the retained intron 6, causing the synthesis of a protein containing 205 amino acids encoded by exons 2, 3, 4, 5, and 6 and an additional 36 amino acids (a.a.) encoded by intron 6 (this protein was named GAS2mu, Fig. 1E). In accordance with the guidelines of the American College of Medical Genetics and Genomics (ACMG) for sequence variant interpretation [17], *GAS2* c.616–2 A > G was interpreted as a pathogenic variant.

### The identified GAS2 variant promotes GAS2 protein degradation via the ubiquitin–proteasome pathway

**Fig. 2:**
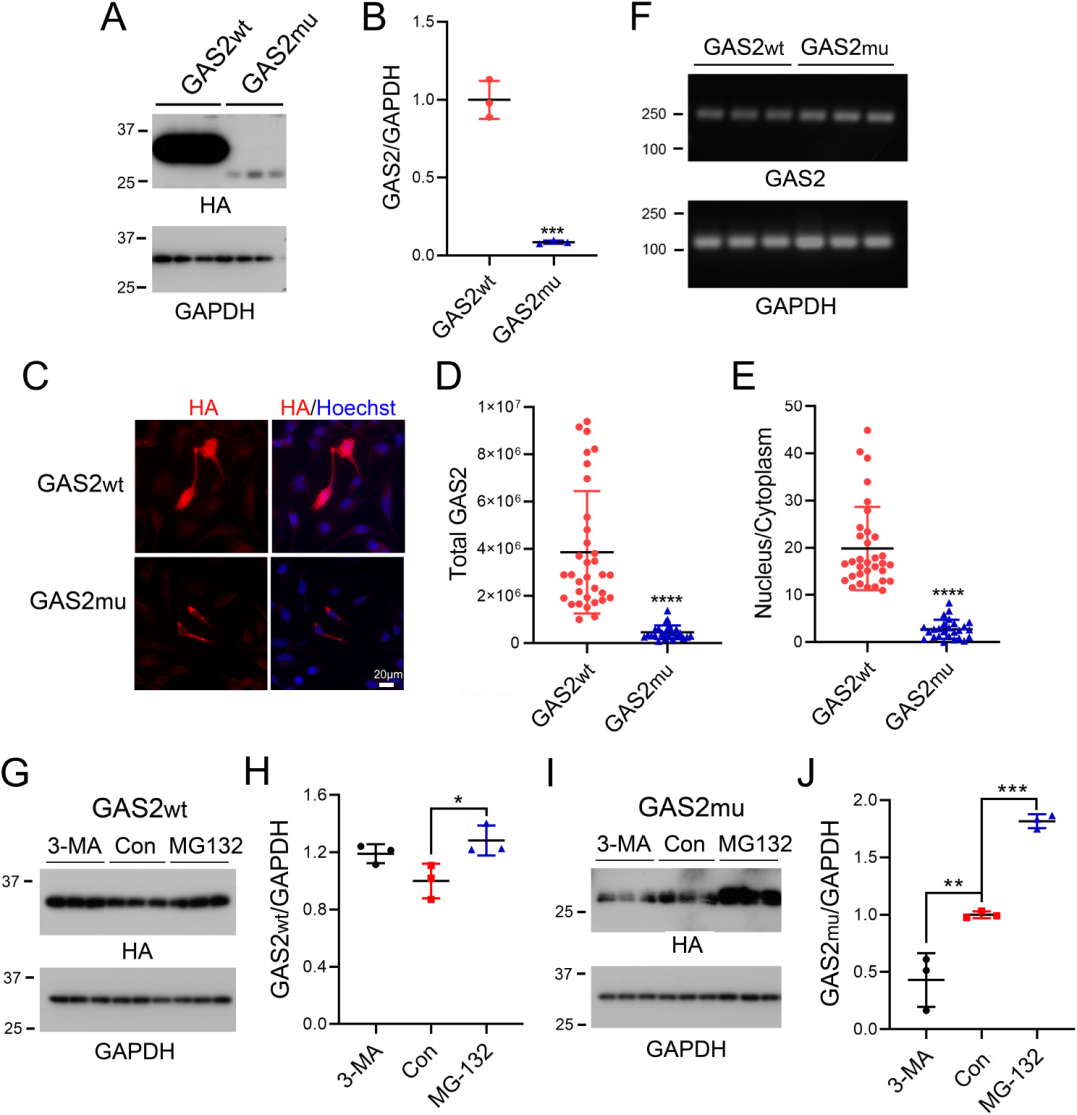
GAS2mu exhibits increased degradation via the ubiquitin–proteasome pathway. (**A-B**) HEK-293T cells were transfected with pCI/HA-GAS2wt or pCI/HA-GAS2mu and were harvested for Western blot analysis with polyclonal anti-HA and anti-GAPDH antibodies (**A**). The protein level of GAS2 was normalized to that of GAPDH (**B**). (**C-E**) GAS2wt and GAS2mu were overexpressed in HeLa cells and immunostained with a monoclonal anti-HA antibody followed by an Alexa Fluor 555-conjugated anti-mouse secondary antibody and the nuclear dye Hoechst 33,342 (**C**). The fluorescence intensity of HA in the nucleus and the cytoplasm was measured by ImageJ. The total cellular fluorescence signal (**D**) and the ratio of nuclear fluorescence to cytoplasmic fluorescence (**F**) were calculated. (**F**) HEK-293T cells were transfected with pCI/HA-GAS2wt or pCI/HA-GAS2mu and harvested for RNA isolation, and the mRNA and GAPDH levels were measured via RT‒PCR. (**G-J**) HEK-293T cells overexpressing GAS2wt (**G**) or GAS2mu (**I**) were treated with 3-MA or MG132 for 12 h, and the protein level of GAS2 was measured via Western blotting (**G, I**) and normalized to that of GAPDH (**H, J**). *n* = 3. The data are presented as the means ± SDs; *, *p* < 0.05; **, *p* < 0.01; ***, *p* < 0.001; and ****, *p* < 0.0001

Given the previous report of another homozygous variant in *GAS2* cosegregating with hearing loss in a family of Somalian descent [5], we speculated that the *GAS2* c.616–2 A > G variant identified herein may be a cause of the progressive, late-onset hearing loss in Family NT33. To investigate the effect of this pathogenic mutation on the function of the associated protein, we constructed plasmids expressing GAS2wt and GAS2mu with an N-terminal HA tag and transfected them into HEK-293T cells. The expression of the GAS2mu was barely detectable and was significantly lower than that of GAS2wt, as shown by Western blotting with an anti-HA antibody (Fig. 2A and B). Consistent with the Western blot results, the immunofluorescence intensity of GAS2mu was also much lower than that of GAS2wt in HeLa cells (Fig. 2C and D). Furthermore, compared with GAS2wt, GAS2mu was localized mainly in the cytoplasm instead of being diffusely distributed in the cell (Fig. 2C and E). However, the mRNA level of *GAS2* was comparable between GAS2wt- and GAS2mu-overexpressing cells, indicating that this variant has no impact on *GAS2* mRNA expression (Fig. 2F). We next assessed the protein stability of GAS2mu. HEK-293T cells overexpressing GAS2wt or GAS2mu were treated separately with the autophagic degradation inhibitor 3-MA or the ubiquitin‒proteasome degradation inhibitor MG-132. In contrast to the protein expression of GAS2wt, the protein expression of GAS2mu was markedly increased in cells treated with MG-132 (Fig. 2G and J). However, cells treated with 3-MA did not exhibit an increase in the protein level of either form of GAS2 (Fig. 2E and F). Taken together, these results suggested that this mutation in GAS2 could efficiently increase protein degradation via the ubiquitin–proteasome pathway rather than the autophagy pathway.

### GAS2mu could not colocalize with microtubule bundles or cause microtubule disorganization

**Fig. 3:**
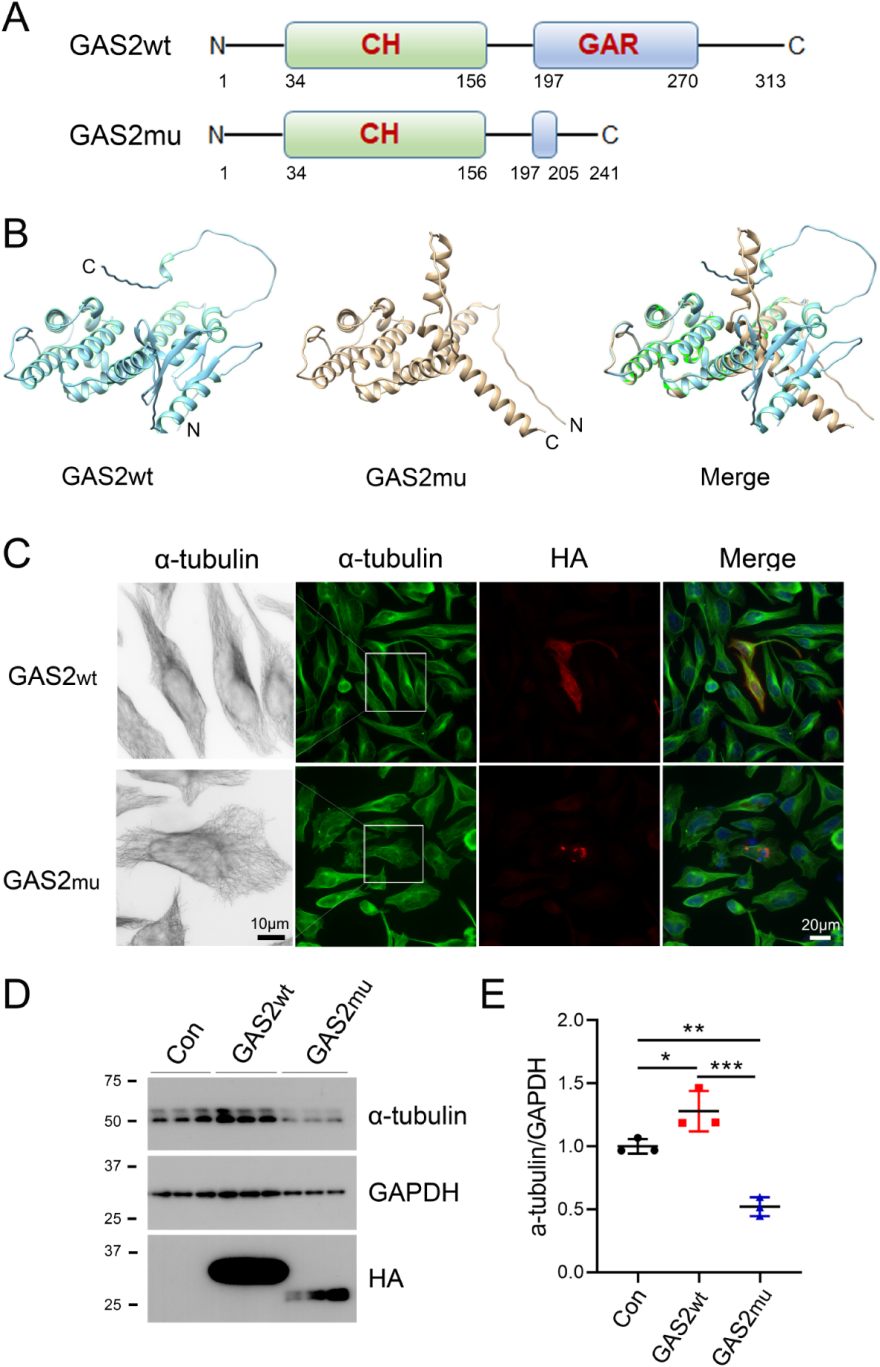
GAS2mu suppresses the protein expression of α-tubulin and causes microtubule disorganization. (**A**, **B**) Schematic diagrams of the GAS2 proteins (**A**) and their structures as determined by protein modeling using AlphaFold 2 (**B**). (**C**) HA/GAS2wt and HA/GAS2mu were overexpressed in HeLa cells and immunostained with anti-HA and anti-α-tubulin antibodies followed by fluorescently labeled anti-rabbit (green) and anti-mouse (red) secondary antibodies. (**D**, **E**) HEK-293T cells overexpressing HA/GAS2wt or HA/GAS2mu were harvested and subjected to Western blot analysis with anti-GAPDH, anti-HA and anti-α-tubulin antibodies (**D**). The ratio of α-tubulin to GAPDH was calculated, and significance was analyzed by paired Student’s t test (**E**). *n* = 3. The data are presented as the means ± SDs; *, *p* < 0.05; **, *p* < 0.01; and ***, *p* < 0.001

GAS2wt is a protein of 313 a.a. that contains two domains, i.e., a CH domain and a GAR domain, while GAS2mu is a C-terminally truncated protein of 241 a.a. that contains only a CH domain (Fig. 3A). We then predicted the protein structures of GAS2wt and GAS2mu using AlphaFold2 [13]. The structures of the N-terminus were consistent between GAS2wt and GAS2mu (Fig. 3B). Notably, the C-terminus of GAS2wt contained five β-sheets and one α-helix, but the C-terminus of GAS2mu contained only two α-helices (Fig. 3B), suggesting that this novel variant may disrupt the binding of the encoded protein with microtubules. GAS2 localizes to the microtubules of supporting cells in the postnatal cochlea and provides mechanical stiffness to transmit sound energy through the cochlea [5]. Consistent with these observations, we found that GAS2wt was evenly distributed throughout the cell and colocalized with α-tubulin, which is also consistent with its biological function as a cross-linker between microtubules and actin filaments. The microtubules were tightly bundled in GAS2wt cells, as determined by staining with an anti-α-tubulin antibody (Fig. 3C). However, GAS2mu staining was characterized by a punctate pattern instead of an even distribution in the cell, and GAS2mu overexpression caused the microtubules to become disorganized and less tightly bundled (Fig. 3C). Moreover, the protein level of α-tubulin was dramatically increased in the GAS2wt group **(**Fig. 3D and E**)**. However, GAS2mu overexpression significantly attenuated the protein level of α-tubulin, consistent with the sparse microtubule structure **(**Fig. 3D and E**)**. These results demonstrate that the GAS2mu affects the protein stability of GAS2 and perturbs its binding to microtubules.

### The identified GAS2 variant exacerbates apoptosis by increasing the Bcl-xS/Bcl-xL ratio

**Fig. 4:**
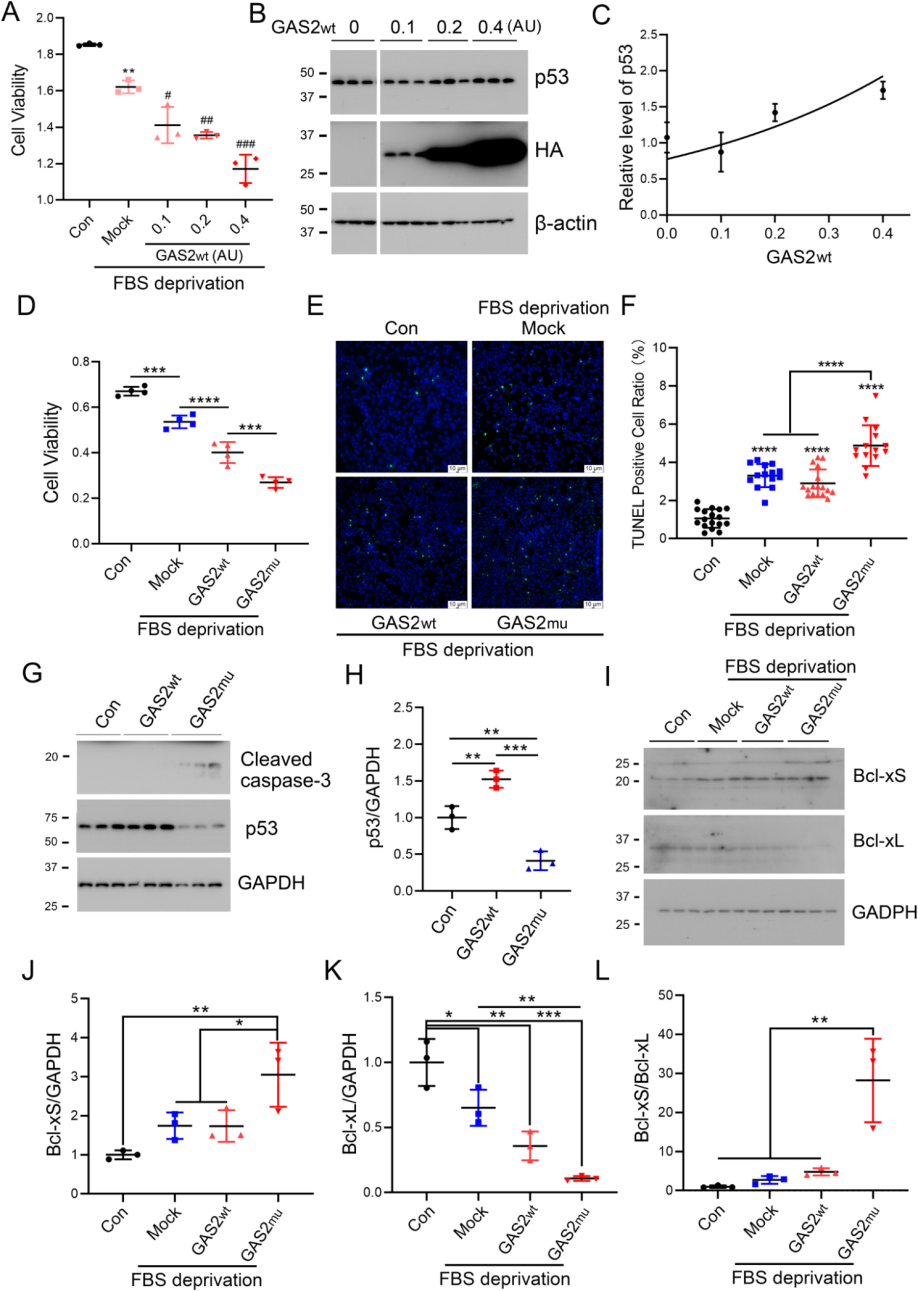
GAS2mu promotes apoptosis by increasing the Bcl-xS/Bcl-xL ratio. (**A**) HEK-293T cells transfected with different amounts of the GAS2wt overexpression plasmid were treated with normal medium or FBS-free medium for 24 h, and cell viability was measured by a CCK8 assay. **(B, C)** HEK-293T cells were transfected with different amounts of the GAS2wt overexpression plasmid and deprived of FBS for 24 h. The protein levels of p53, GAS2 (HA) and β-actin were measured via Western blotting (**B**). The protein level of p53 against the amount of transfected GAS2wt plasmid was visualized (**C**). AU: artificial unit. *: compared with Con, #: compared with Mock. (**D**) HEK-293T cells were transfected with pCI/HA-GAS2wt or pCI/HA-GAS2mu and treated with or without FBS deprivation for 24 h. Cell viability was analyzed via a CCK8 assay. (**E**) Apoptosis was analyzed by a TUNEL assay. (**F**) The number of TUNEL-positive cells was normalized to the total number of cells. (**G**) The protein levels of c-caspase-3, p53, and GAPDH were measured via Western blotting. (**H**) The protein level of p53 was normalized to that of GAPDH. (**I-L**) The protein levels of Bcl-xS and Bcl-xL were measured via Western blotting (**I**), and the Bcl-xS/GAPDH (**J**), Bcl-xL/GAPDH (**K**) and Bcl-xS/Bcl-xL (**L**) ratios were calculated. The data are presented as the means ± SDs; *, *p* < 0.05; **, *p* < 0.01; ***, *p* < 0.001; and ****, *p* < 0.0001

Abnormally excessive cellular apoptosis in the inner ear has also been observed and determined to play a vital role in progressive ADNSHL [19, 24]. Therefore, we speculated that GAS2mu might cause progressive late-onset hearing loss via apoptosis. Overexpression of GAS2wt dose dependently decreased cell viability under FBS deprivation conditions via upregulation of p53 (Fig. 4A and C). In addition, compared with overexpression of GAS2wt, overexpression of GAS2mu further reduced cell viability (Fig. 4D) and increased susceptibility to apoptosis, as indicated by the increased TUNEL-positive rate (Fig. 4E and F) and increased c-caspase 3 protein level (Fig. 4G) under FBS deprivation conditions in HEK-293T cells. However, unlike in GAS2wt cells, p53 expression was downregulated in GAS2mu cells under FBS deprivation conditions (Fig. 4G and H), revealing that GAS2mu may promote apoptosis in a p53-independent manner. Bcl-x has two isoforms: the antiapoptotic isoform (Bcl-xL) and the proapoptotic isoform (Bcl-xS). Overexpression of Bcl-xL prevents cochlear hair cell death due to aminoglycoside ototoxicity and hearing loss in mice [14, 15]. Thus, we further investigated the effect of GAS2mu on the expression of Bcl-xS and Bcl-xL. We found that overexpression of GAS2wt had no impact on either the Bcl-xS or the Bcl-xL level in FBS-deprived cells (Fig. 4I). However, overexpression of GAS2mu dramatically increased the level of the proapoptotic isoform Bcl-xS and decreased the level of the antiapoptotic Bcl-xL isoform (Fig. 4I and L), indicating that GAS2mu promotes apoptosis by regulating the alternative splicing of Bcl-x. Thus, our results demonstrated that GAS2mu exacerbated apoptosis by increasing the ratio of Bcl-xS to Bcl-xL under serum deprivation conditions instead of promoting apoptosis in a p53-dependent manner.

**Fig. 5:**
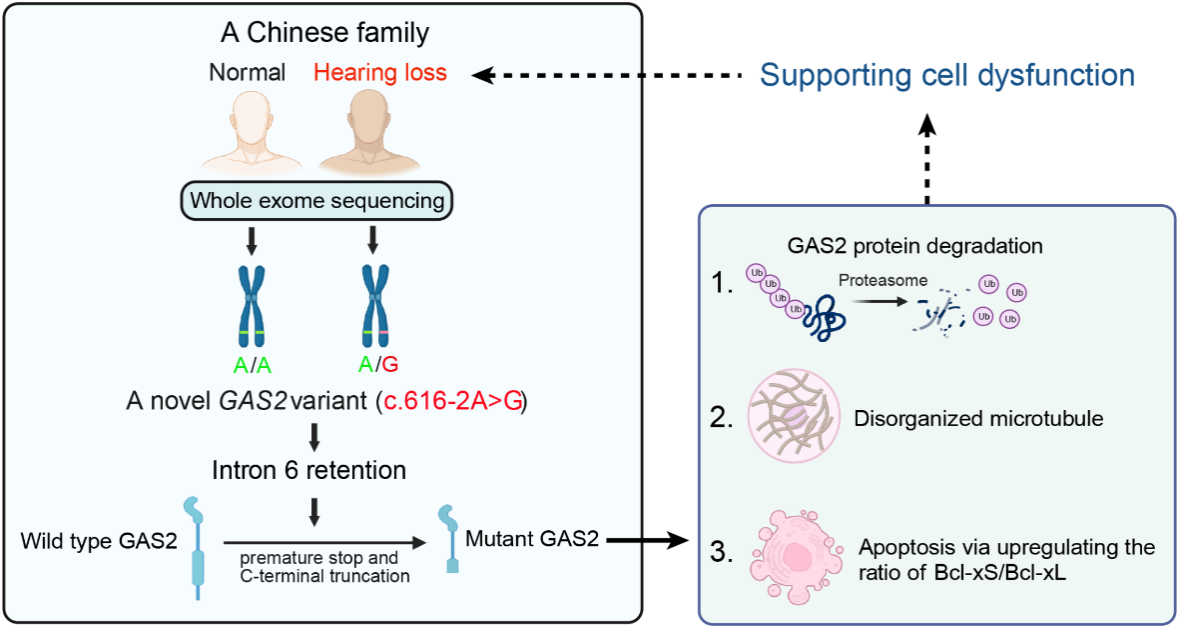
Schematic showing the molecular mechanism by which a novel *GAS2* variant leads to late-onset, progressive NSHL in a Chinese family. By WES, we identified a novel heterozygous *GAS2* variant (c.616–2 A > G) segregating with nonsyndromic hearing loss in a dominant family. This variant caused the retention of intron 6 in the mature GAS2 mRNA, leading to an in-frame stop codon in the retained intron 6 and the synthesis of a truncated GAS2 protein. Compared with the wild-type GAS2 protein, the truncated GAS2 protein exhibited aggregation and increased degradation via the proteasome, and cells expressing this protein exhibited microtubule disorganization, cytoskeletal abnormalities, and increased apoptosis, which was mediated via an increase in the Bcl-xS/Bcl-xL ratio. The expression and function of GAS2 in supporting cells have previously been reported. Thus, we assumed that the pathological function of the truncated GAS2 protein reported in this study might result in cytoskeletal abnormalities and increased apoptosis in supporting cells, leading to hearing loss in affected individuals. The schematic was created by BioRender (https://www.biorender.com/).

Taken together, the identified splicing variant in *GAS2*, associating with ADNSHL, leads to an intron retention, the formation of a premature stop codon and the synthesis of a C-terminally truncated GAS2 protein with loss of the microtubule-binding domain. Our results revealed that this mutation induces GAS2 protein degradation through the ubiquitin-proteasome pathway, severely disrupts microtubule organization and exacerbates apoptosis by upregulating the expression of Bcl-xS. These mechanisms may underlie the impact of *GAS2* variant on inner ear supporting cell dysfunction, ultimately contributing to hearing loss (Fig. 5).

## Discussion

Herein, we identified a novel heterozygous pathogenic variant in *GAS2* (MIM No. 600,840) that cosegregated with ADNSHL in a Chinese family. To our knowledge, this is the first reported splicing variant in *GAS2* that is associated with ADNSHL. We also investigated the effect of this novel *GAS2* variant on the biological function of the encoded protein to illustrate its pathogenic effect for the first time.

GAS2 consists of a CH domain and a GAR domain, which allow GAS2 to function as a cross-linker between actin filaments and microtubules, respectively. The truncated GAS2 protein identified herein retained only the first eight a.a. (197–205) of the GAR domain. Unlike GAS2wt, GAS2mu formed aggregates and lost the ability to colocalize with α-tubulin. A previous study demonstrated that artificial deletion of the C-terminus of GAS2 as, for example, in the GAS2_Δ276−314_ and GAS2_Δ236−314_ mutants, resulted in changes in cell morphology [3]. Moreover, further deletion of C-terminal residues, as in the GAS2_Δ200−314_ and GAS2_Δ171−314_ mutants, resulted in alterations in the microfilament system without alterations in cell morphology [3]. Consistent with this observation, we also found that GAS2mu had no obvious effect on cell morphology. Hyperphosphorylation and truncation of tau, a microtubule-associated protein, facilitate its aggregation [9]. Like truncated tau, GAS2mu could also be prone to self-aggregation, and this possibility should be further investigated via cellular and biochemical experiments.

Under stress conditions, such as FBS deprivation and exposure to DNA-damaging agents, GAS2 increases cell susceptibility to p53-dependent apoptosis by inhibiting calpain activity [2]. Subsequently, activated caspase-3 and caspase-7 can cleave their death substrate GAS2. The cleaved form of GAS2 induces morphological changes during cellular apoptosis [18]. We also found that overexpression of GAS2wt promoted apoptosis and increased p53 expression under FBS deprivation conditions. In contrast, the dominant-negative form of GAS2 (GAS2_Δ171−314_) cannot prevent p53 degradation by calpain, leading to decreased susceptibility to apoptosis [2]. Strikingly, in contrast to GAS2_Δ171−314_, GAS2mu increased cellular susceptibility to apoptosis upon FBS deprivation. These findings indicate that GAS2mu gains the function of promoting apoptosis. Deficiency of *Thoc1*, an ARHL risk gene, induces the expression of proapoptotic genes and results in hair cell apoptosis, leading to ARHL in patients [24]. Furthermore, we showed that the increase in apoptosis induced by GAS2mu was associated with an increase in the Bcl-xS/Bcl-xL ratio. Alternative splicing of Bcl-x exon 2b results in the production of two isoforms of Bcl-x: the pro-apoptotic form Bcl-xS and the anti-apoptotic form Bcl-xL [27]. Therefore, we revealed a distinct molecular mechanism underlying the enhancement of apoptosis by GAS2mu relative to GAS2wt. These findings may provide additional insights into the pathological roles of this novel *GAS2* variant in hearing loss.

More than 80% of protein degradation in mammalian cells is catalyzed by the 26 S proteasome. Misfolded and unusable proteins are first ubiquitinated and are subsequently transferred to the proteasome to be digested for protein turnover, which is essential for protein homeostasis [6]. Here, we found that GAS2mu not only lost its physiological function but also became a toxic protein that increased apoptosis. Consequently, GAS2mu was degraded via the proteasome, resulting in a reduction in its protein level. We assume that the affected members of this Chinese family exhibit lower GAS2 protein expression levels than healthy individuals, a characteristic that may indicate another pathological function of this novel *GAS2* variant in hearing loss.

GAS2 localizes with microtubules and regulates microtubule stability and organization in the supporting cell of the postnatal cochlea [5]. GAS2 maintains the stiffness properties of the cochlear supporting cells for the propagation and amplification of travelling waves via the cochlear partition in response to sound. Two *GAS2* variants, c.616-2A > G (Chinese family in this study) and c.723 + 1G > A (Somalian family), locating at the 3’ splicing site and 5’ splicing site of the same exon respectively, both caused the C-terminal truncation of GAS2 protein and associated with the hearing loss. For the mechanisms regarding two different *GAS2* variants leading to hearing loss, we speculate that the proteins encoded by these two *GAS2* variants have partial GAR domain, especially for the c.616–2 A > G which produces a shorter GAR domain. The GAR domain plays a vital role in mediating the interaction between GAS2 and microtubules [26]. So, the proteins encoded by these two variants may totally or partially lose their interaction with microtubules in the supporting cell of the postnatal cochlea. Furthermore, the two variants both could cause the retention of intron. These additional amino acids encoded by the intron may endow a novel biological function or characteristic for the GAS2 mutants. As shown in this study, GAS2mu promotes its own protein degradation and cellular apoptosis, which endows the novel variant gaining negative biological functions. Thus, we speculated that these additional amino acids might also contribute to the dysfunction of supporting cells.

In summary, we not only identified a novel *GAS2* variant but also expounded on the pathogenic association of this variant with hearing loss in vitro. Our study not only expands the landscape of pathogenic genetic variants but also provides novel insights into the genetic basis of hearing loss, which will greatly contribute to the molecular diagnosis of hearing loss and therapeutic developments for this condition. A limitation of this study is that the genetic basis of GAS2mu was explored only in vitro, and further investigations should be conducted in vivo to further elucidate the pathogenic roles of GAS2 mutants in hearing loss.

### Electronic supplementary material

Below is the link to the electronic supplementary material.

Supplementary Material 1

## Data Availability

The datasets provided in this study are available in online repositories.

## Abbreviations

GAS2: Growth Arrest Specific Protein 2
CH: Calponin Homology
GAR: Growth Arrest Specific Related
GAS2wt: Wild-Type GAS2
GAS2mu: Truncated GAS2
WES: Whole-Exome Sequencing
HL: Hearing Loss
ADNSHL: Autosomal Dominant Nonsyndromic Hearing Loss
PTA: Pure Tone Audiometry
FBS: Fetal Bovine Serum
CCK8: Cell Counting Kit-8
OD: Optical Density
ACMG: American College of Medical Genetics and Genomics
