# Supplementary figures and images for: A novel variant in GAS2 is associated with autosomal dominant nonsyndromic hearing impairment in a Chinese family
Source: Hum Genomics. 2024 Jul 2;18:73. doi: 10.1186/s40246-024-00628-2 (PMC11218307; doi:10.1186/s40246-024-00628-2)

**Supplementary material:**

**
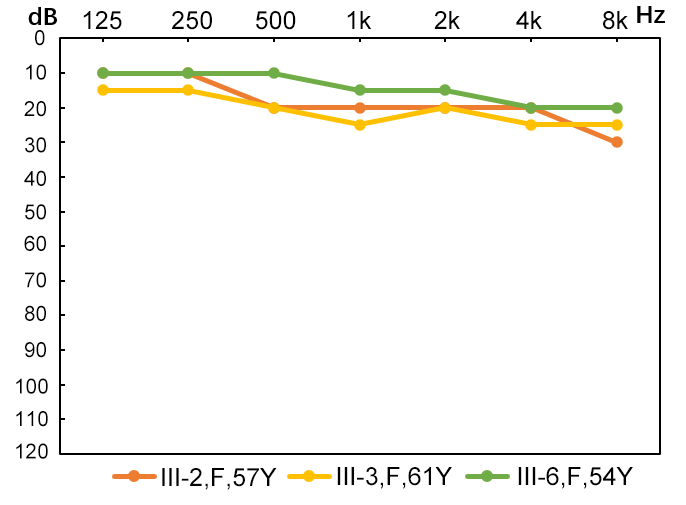
**

**Fig. S1. Representative audiograms of normal members of Family NT33.**

Supplement: Supplementary file 1 — Supplementary Material 1 [file 40246_2024_628_MOESM1_ESM.docx]
